# Lack of evidence for effects of lockdowns on stillbirth rates during the SARS-CoV-2 pandemic in Bavaria: analysis of the Bavarian perinatal survey from 2010 to 2020

**DOI:** 10.1007/s00404-022-06838-0

**Published:** 2022-11-09

**Authors:** Florian Matthias Stumpfe, Michael Oliver Schneider, Sophia Antoniadis, Andreas Mayr, Tobias Fleckenstein, Christian Staerk, Sven Kehl, Peter Hermanek, Julian Böhm, Anton Scharl, Matthias Wilhelm Beckmann, Alexander Hein

**Affiliations:** 1 Department of Obstetrics and Gynecology, Friedrich-Alexander-Universität Erlangen-Nürnberg, University Hospital of Erlangen, Universitätsstraße 21-23, 91054 Erlangen, Germany; 2 Department of Medical Biometry, Informatics and Epidemiology, Faculty of Medicine, University of Bonn, Bonn, Germany; 3 Department of Methods and Perinatology, BAQ, Bavarian Institute for Quality Assurance, Munich, Germany; 4 Klinik Bad Trissl, Oberaudorf, Germany

**Keywords:** COVID-19, Stillbirths, Stillborn

## Abstract

**Background:**

Internationally, potential effects of national SARS-CoV-2-related lockdowns on stillbirth rates have been reported, but data for Germany, including risk factors for fetal pregnancy outcome, are lacking. The aim of this study is to compare the stillbirth rates during the two first lockdown periods in 2020 with previous years from 2010 to 2019 in a large Bavarian cohort.

**Methods:**

This study is a secondary analysis of the Bavarian perinatal data from 2010 to 2020, including 349,245 births. Univariate and multivariable regression analyses were performed to investigate the effect of two Bavarian lockdowns on the stillbirth rate in 2020 compared to the corresponding periods from 2010 to 2019.

**Results:**

During the first lockdown, the stillbirth rate was significantly higher compared to the reference period (4.04 vs. 3.03 stillbirths per 1000 births; *P* = 0.03). After adjustment for seasonal and long-term trends, this effect can no longer be observed (*P* = 0.2). During the second lockdown, the stillbirth rate did not differ in univariate (3.46 vs. 2.93 stillbirths per 1000 births; *P* = 0.22) as well as in multivariable analyses (*P* = 0.68), compared to the years 2010 to 2019.

**Conclusion:**

After adjustment for known long-term effects, in this study we did not find evidence that the two Bavarian lockdowns had an effect on the rate of stillbirths.

**Supplementary Information:**

The online version contains supplementary material available at 10.1007/s00404-022-06838-0.

## What does this study add to the clinical work?

**Table:** 

In this study, after adjusting for long-term trends we did not find evidence that lockdowns during the SARS-CoV-2 pandemic had an impact on stillbirth rates in Bavaria.	

## Introduction

Following the discovery of the new SARS-CoV-2 virus, different infection control measures—including lockdowns with curfews—were introduced in many countries to control infection rates.

Especially the possible impact of an infection during pregnancy has been discussed and studied extensively among experts [1, 2]. In addition, it has also been questioned whether lockdown periods could possibly influence pregnancy outcome. For example, a report from Nepal showed that a reduced number of prenatal check-ups resulted in an increased stillbirth rate [3].

Further international studies from London and Italy also reported increased stillbirth rates associated with lockdown periods [4, 5]. However, in other countries—e.g. Spain or USA—such an increase was not observed [6, 7]. While health care in low-income and high-income countries is essentially different and thus a comparison is difficult, there are also considerable differences between high-income countries. Therefore, existing data on the possible impact of lockdowns on the stillbirth rate, e.g. from the UK, are not representative for Germany. Hence, the aim of this study was to provide evidence for Germany based on the Bavarian Perinatal Survey.

## Methods

### Study design

This is a secondary analysis of Bavarian perinatal birth census data. It is based on the requirement that data on all births must be reported centrally according to defined criteria. Following a request, the data set of the reported deliveries was made available to our working group in anonymised form for evaluation by the Bavarian Working Group for Quality Assurance in Inpatient Care (Bayerische Arbeitsgemeinschaft für Qualitätssicherung in der stationären Versorgung, BAQ).

### Setting and study period

In Bavaria, various measures were established by the Bavarian government to mitigate the incidence of SARS-CoV-2. These measures were adopted to the infection numbers and ranged from general hygiene measures up to curfews. An overview of infection control measures in Bavaria is shown in a previous paper by our study group [8].

Based on these infection control measures, two lockdown periods were defined for the year 2020, which accordingly represent the study periods:

- Lockdown period I: March 16th, 2020 to May 6th, 2020.
- Lockdown period II: November 2nd, 2020 to December 31st, 2020.

It should be noted that the second infection wave continued into the year 2021 and merged into the third wave; thus, the studied period of lockdown period II ends with December 31st, 2020.

### Study cohort

In this study, stillbirths ≥ 24 + 0 weeks of gestation during both lockdown periods were analysed. Data on miscarriages and pregnancy terminations < 24 + 0 weeks of gestation were obtained from the public database of the Federal Statistical Office [9].

### Ethical vote

As this is an analysis of centrally collected, anonymised data, no additional ethics vote was required for this study.

### Statistical analyses

The exploratory data analysis was initially conducted descriptively by comparing the characteristics of stillbirths during the lockdown periods in 2020 with those in the corresponding periods from 2010 to 2019 (numbers and percentages). To test for possible associations, the odds ratio (OR) was determined as a measure of association between binary variables (e.g. stillbirth yes/no and year 2020/2010–2019) with associated 95% confidence interval and *P* value. Finally, to adjust for possible seasonal (cyclic spline for calendar weeks) and known long-term trends (year), logistic generalised additive models were estimated over all births from 2010 to 2020. The occurrence of a stillbirth served as the binary response variable, with year, month and day of the week being adjusted for via penalised non-linear splines (cyclical splines for month and day of the week). In addition, indicator variables marked the lockdown periods in 2020, from whose coefficients adjusted odds ratios (adj. OR) were then determined, again with 95% confidence intervals and *P* values. These analyses were repeated separately for various characteristics (gestational age, timing, sex of the child) within the subgroup of stillbirths. A *P* value < 0.05 was considered statistically significant; no adjustment for multiple testing was considered due to the exploratory nature of the analysis. All analyses were performed within the statistical computing environment R (Version 4.1.2) using corresponding add-on packages for the additive regression models [10].

## Results

Overall, 349,245 births were provided for this study. Of these, 314,943 births were reported for the years 2010–2019 and 34,302 births for 2020. During the first lockdown period, 16,080 births were reported in Bavaria in 2020. During the corresponding period from 2010 to 2019, there were 145,458 births. Respectively, during the second lockdown period, there were 18,222 births in 2020 and 169,485 births in the corresponding period.

Table 1 shows descriptive data of cases with stillbirth during the first lockdown period. Similarly, Table 1S shows the descriptive data for the second lockdown period (supplement). With regard to maternal age and number of previous miscarriages, no difference could be demonstrated compared to the corresponding study period of 2010–2019 during the first lockdown. There were significantly more cases with stillbirths with three or more miscarriages.

**Table 1:** Characteristics of the study population with stillbirth in the first lockdown compared to the corresponding period from 2010 to 2019

	2020	2010–2019	OR (95% CI)	*P* value
Total number of stillbirths (*n*, %)	65 (100)	440 (100)		
Maternal age; years (*n*, %)				
18–22	5 (7.7)	36 (8.2)	0.94 (0.28–2.35)	1.00
23–27	10 (15.4)	75 (17)	0.89 (0.38–1.86)	0.86
28–32	21 (32.3)	139 (31.6)	1.03 (0.56–1.86)	0.89
33–37	19 (29.2)	119 (27.0)	1.11 (0.59–2.03)	0.77
≥ 38	10 (15.4)	66 (15.0)	1.03 (0.45–2.17)	1.00
Gravidity (*n*, %)				
0	20 (30.8)	172 (39.1)	0.69 (0.37–1.25)	0.22
1	17 (26.2)	125 (28.4)	0.89 (0.46–1.65)	0.77
2	9 (13.8)	79 (18.0)	0.73 (0.31–1.58)	0.49
≥ 3	19(29.2)	64 (14.5)	2.42 (1.26–4.53)	**0.006**
Previous miscarriages (*n*, %)				
0	44 (67.7)	255 (58.0)	2.26 (0.32–97.43)	0.70
1	1 (1.5)	13 (3.0)	0.45 (0.01–3.12)	0.70
≥ 2	0 (0.00)	0 (0.00)	0.0	1.00
Not specified	20 (30.8)	172 (39.1)	1.44 (0.37–1.25)	0.22
Pregnancy risks (*n*, %)				
Diabetes mellitus	12 (18.5)	40 (9.1)	2.26 (1.01–4.74)	**0.03**
Gestational diabetes	5 (7.7)	16 (3.6)	2.20 (0.61–6.60)	0.17
Arterial hypertension	3 (4.6)	7 (1.6)	2.98 (0.49–13.5)	0.13
Drug abuse	0 (0.0)	10 (2.3)	0.00 (0.00–3.03)	0.37

Bold symbols means statistical signicant

The absolute number and the percentage of all stillbirths in the corresponding time period are shown

*OR* odds ratio *n* numbers

Regarding pregnancy risks, no significant difference in the incidence of gestational diabetes, gestational hypertension and drug abuse could be detected, whereas the proportion of patients with pre-existing diabetes mellitus was twice as high in the first lockdown than in the corresponding period (18.5 vs. 9.1%; *P* = 0.03).

During the second lockdown, there were more cases with stillbirth and maternal age ≥ 38 years.

(25.4 vs. 12.7%; *P* = 0.01). No other characteristics differed significantly.

In the univariate analysis, the stillbirth rate was significantly higher during the first lockdown period compared to the corresponding period from 2010 to 2019 (Table 2). In 2020, the stillbirth rate was 4.04 per 1000 total births, while in the control period it was 3.03 per 1000 total births, which was significantly lower (OR 1.3; *P* = 0.03). During the second lockdown period, the stillbirth rate was not significantly different (3.46 vs. 2.93; *P* = 0.22) (Table 2S).

**Table 2:** Details on stillbirths in Bavaria during the first lockdown compared to the corresponding period from 2010 to 2019

	2020	2010–2019	OR (95% CI)	*P* value	Adj. OR (95% CI)	Adj. *P* value
Total number of births during lockdown (*n*)	16080	145 458				
Stillbirths (*n*)	65	440				
Stillbirths per 1000 total births	4.04	3.03	1.3 (1–1.7)	**0.03**	1.2 (0.9–1.6)	0.2
Gestational age at stillbirth (weeks + days)						
24 + 0 to 25 + 6	5 (7.7)	48 (10.9)	0.68 (0.20–1.80)	0.52	0.69 (0.27–1.78)	0.44
26 + 0 to 27 + 6	8 (12.3)	38 (8.6)	1.48 (0.57–3.44)	0.35	1.33 (0.61–2.90)	0.48
28 + 0 to 31 + 6	8 (12.3)	56 (12.7)	0.96 (0.38–2.17)	1.00	0.76 (0.36–1.62)	0.48
32 + 0 to 36 + 6	14 (21.5)	106 (24.1)	0.87 (0.43–1.67)	0.76	0.83 (0.44–1.56)	0.57
37 + 0 to 40 + 0	9 (13.8)	62 (14.1)	1.12 (0.56–2.14)	0.75	0.94 (0.45–1.95)	0.87
> 40 + 0	5 (7.7)	32 (7.3)	1.06 (0.31–2.90)	0.80	1.11 (0.43–2.93)	0.83
Timing of stillbirth						
Antepartum	41 (63.1)	281 (63.9)	0.97 (0.55–1.74)	0.89	0.87 (0.50–1.52)	0.63
Intrapartum	5 (7.7)	31 (7.0)	1.10 (0.32–3.01)	0.80	1.20 (0.46–3.12)	0.71
Not specified	19 (29.2)	128 (29.1)	1.01 (0.54–1.83)	1.00	0.93 (0.53–1.63)	0.79
Foetal sex						
Female	34 (52.3)	237 (53.9)	0.94 (0.54–1.64)	0.89	1.11 (0.66–1.88)	0.69
Male	31 (47.7)	203 (46.1)	1.06 (0.61–1.86)	0.89	0.90 (0.53–1.52)	0.69

Bold symbols means statistical signicant

*OR* odds ratio, *CI* confidence interval

Since the number of stillbirths in Bavaria increased since 2012, an adjustment for long-term trends and seasonal effects was performed in a subsequent step. After adjustment, no significant effect of the first lockdown on stillbirth rates could be demonstrated (*P* = 0.1).

Similarly, there is no evidence for an effect of the second lockdown period on the stillbirth rate (*P* = 0.68). With regard to abortions below 24 + 0 weeks of gestation, the increasing trend which has been observed since 2012 can be observed (supplement Table 3S).

## Discussion

In the present study, we could not prove any impact of both lockdown periods in 2020 on the stillbirth rate during the SARS-CoV-2 pandemic in Bavaria. This is in line with study results from other countries: in Botswana—an upper middle-income country—the rate of stillbirths during the lockdown period from April to May 2020 remained stable compared to the corresponding period from 2017 to 2019 [11]. In the UK—a high-income country—also no increase of stillbirths could be demonstrated. The authors of the UK study [12] compared the lockdown period from April to June 2020 with the corresponding period in 2019 (0.41 vs. 0.40%; *P* = 0.69). Interestingly—while there were not more stillbirths in UK—a significantly higher rate of stillbirths was reported in the City of London [5]. The authors of the London study speculate that women may have been more hesitant to go to the clinic than before the pandemic to avoid infection with SARS-CoV-2. They also speculate whether asymptomatic SARS-CoV-2 infections could be the cause of the increased stillbirth rate. In their study population, there were no cases with confirmed SARS-CoV-2 infection.

Increased stillbirth rates were not just reported in the London metropolitan area. A retrospective single centre cohort study from Tel Aviv, Israel, also demonstrated a significantly higher stillbirth rate (0.4 vs. 0.1%; *P* = 0.04)—though in a small number of cases [13]. An Italian study from the Lazio region provides a considerably higher number of cases. Also in this Italian region, which includes the capital Rome, significantly more stillbirths were observed during the lockdown from March to May 2020 compared to the same period in 2019 (3.23 vs. 1.07%; *P* = 0.0017) [4]. An increase in stillbirths was also reported from Nepal—in this context, the authors also report an evident decline in institutional deliveries. A reduced number of preventive check-ups is suspected. This could explain the increased rate of pregnancy complications in the studied period [3]. However, it remains unclear whether the difference to our results can also be explained by the number of screening examinations in our study population, as this is not an obligatory parameter to be reported within the quality assurance process.

One indication for a reduced perception of medical check-ups could be that patients with pre-existing diabetes mellitus had a significantly higher incidence of stillbirth in 2020. It can be hypothesised that the blood glucose control of these patients was less adequate during the SARS-CoV-2 pandemic than in the previous years due to reduced diabetological monitoring. However, at the beginning of the SARS-CoV-2 pandemic, there were various statements on the relevance of prenatal care. To what extent these had an impact on the adherence to screening intervals remains unclear. Due to the stable stillbirth rate, it can be assumed that prenatal care services remained available. Regarding the steady stillbirth rate, our study is in line with a publication on the aggregate data of the Federal Republic of Germany. Kniffka et al. [14] analysed publicly available data from the Federal Statistical Office (Destatis) and were able to show that the stillbirth rate in the first half of 2021 followed the rising trend of previous years, but did not increase significantly.

At first sight, our data suggest that stillbirths were significantly higher during the first lockdown than in previous years. However, after adjustment for long-term effects and seasonal trends, this effect is no longer detectable. This could explain the discrepancy compared to data from other regions of industrial countries that did not adjust accordingly [5]. Furthermore, our study analyses a possible effect of lockdown periods on a significantly higher number of cases than other studies [13, 15] and provides data on baseline data and pregnancy risks in patients with stillbirths. Since there is no information available from the Federal Statistical Office on causes of stillbirths or maternal diseases, our data thus complement the study by Kniffka [14].

A strength of our study is that we can expand the existing evidence based on defined quality parameters and centrally collected data records using a large number of cases. In addition, we can provide data on the gestational age of stillbirths which have not yet been published for Germany. Since the gestational age at stillbirth does not differ from the reference period, it can be assumed that there was no shift in the time of diagnosis due to the lockdown periods in Bavaria. However, centralised data also have a weakness: the overall quality of the data depends on the individual quality of the reported deliveries. For example, in about a third of the cases the exact time of stillbirth is unknown — despite this stable proportion over the years, this considerably reduces the reliability of the analysed data set.

## Conclusion

In conclusion, we observe no significant influence of both lockdowns in Bavaria on the stillbirth rate after adjustment for long-term trends. Based on a large number of cases, we can thus add further evidence to the existing international debate on this topic.

## Supplementary Information

Below is the link to the electronic supplementary material.

Supplementary file1 (DOCX 21 KB)

Supplementary file2 (DOCX 20 KB)

Supplementary file3 (DOCX 17 KB)

## Data Availability

Data are available on request due to privacy or other restrictions.
