# Supplementary material for: Lack of evidence for effects of lockdowns on stillbirth rates during the SARS-CoV-2 pandemic in Bavaria: analysis of the Bavarian perinatal survey from 2010 to 2020
Source: Arch Gynecol Obstet. 2022 Nov 9;308(5):1457–62. doi: 10.1007/s00404-022-06838-0 (PMC9643984; doi:10.1007/s00404-022-06838-0)
Supplement: Supplementary file 1 — Supplementary file1 (DOCX 21 KB) [file 404_2022_6838_MOESM1_ESM.docx]

|  | **2020** | **2010-2019** | **OR (95% CI)** | **p-value** |
| --- | --- | --- | --- | --- |
| ***Total number of stillbirths*** (n, %) | 63 (100) | 496 (100) |  |  |
| **Maternal age; years** (n, %) | | | | |
| 18-22 | 3 (4.8) | 33 (6.7) | 0.70 (0.13 - 2.34) | 0.79 |
| 23-27 | 9 (14.3) | 93 (18.8) | 0.73 (0.3 - 1.55) | 0.49 |
| 28-32 | 17 (27.0) | 159 (32.1) | 0.78 (0.41 - 1.45) | 0.47 |
| 33-37 | 18 (28.6) | 147 (29.6) | 0.95 (0.5 - 1.74) | 1.00 |
| ≥ 38 | 16 (25.4) | 63 (12.7) | 2.34 (1.2 - 4.50) | **0.01** |
| **Gravidity** (n, %) | | | | |
| 0 | 27 (42.9) | 200 (40.3) | 1.11 (0.63 - 1.95) | 0.79 |
| 1 | 15 (23.8) | 154 (31.0) | 0.69 (0.35 - 1.31) | 0.31 |
| 2 | 9 (14.3) | 77 (15.5) | 0.91 (0.38 - 1.95) | 1.00 |
| ≥ 3 | 12 (19.0) | 65 (13.1) | 1.56 (0.72 - 3.16) | 0.24 |
| **Previous miscarriages** (n, %) | | | | |
| 0 | 35 (55.6) | 287 (57.9) | 1.09 (0.14 - 49.46) | 1.00 |
| 1 | 1 (1.6) | 7 (1.4) | 1.18 (0.03 - 9.63) | 0.61 |
| ≥ 2 | 0 (0.00) | 2 (0.4) | 0.0 (0 - 44.20) | 1.00 |
| Not specified | 27 (42.9) | 200 (40.3) | 0.90 (0.6 - 1.95) | 0.79 |
| **Pregnancy risks** (n, %) | | | | |
| Diabetes mellitus | 12 (19.0) | 63 (12.7) | 1.62 (0.74 - 3.29) | 0.17 |
| Gestational diabetes | 3 (4.8) | 15 (3.0) | 1.60 (0.29 - 5.90) | 0.44 |
| Arterial Hypertension | 0 (0.0) | 7 (1.4) | 0.00 (0 - 5.52) | 1.00 |
| Drug abuse | 4 (6.3) | 17 (3.4) | 1.91 (0.45 - 6.12) | 0.28 |

Table 1S Characteristics of the study population with stillbirth in the second lockdown compared to the corresponding period from 2010-2019.
The absolute number and the percentage of all stillbirths in the corresponding time period are shown.
OR Odds ratio n numbers
