# Supplementary material for: Lack of evidence for effects of lockdowns on stillbirth rates during the SARS-CoV-2 pandemic in Bavaria: analysis of the Bavarian perinatal survey from 2010 to 2020
Source: Arch Gynecol Obstet. 2022 Nov 9;308(5):1457–62. doi: 10.1007/s00404-022-06838-0 (PMC9643984; doi:10.1007/s00404-022-06838-0)
Supplement: Supplementary file 2 — Supplementary file2 (DOCX 20 KB) [file 404_2022_6838_MOESM2_ESM.docx]

|  | **2020** | **2010-2019** | **OR (95% CI)** | **P-value** | **Adj. OR (95% CI)** | **Adj. P-value** |
| --- | --- | --- | --- | --- | --- | --- |
| Total number of births during lockdown (n) | 18 222 | 169 485 |  | | | |
| Stillbirths (n) | 63 | 496 |  |  |  |  |
| Stillbirths per 1000 total births | 3.46 | 2.93 | 1.2 (0.9 - 1.5) | 0.22 | 1.1 (0.8 - 1.4) | 0.68 |
| **Gestational age at stillbirth** (weeks + days) | | | | | | |
| 24+0 to 25+6 | 8 (12.7) | 48 (9.7) | 1.36 (0.53 - 3.10) | 0.50 | 1.18 (0.54 - 2.60) | 0.68 |
| 26+0 to 27+6 | 7 (11.1) | 50 (10.1) | 1.12 (0.41 - 2.64) | 0.82 | 0.99 (0.43 - 2.25) | 0.98 |
| 28+0 to 31+6 | 12 (19.0) | 66 (13.3) | 1.53 (0.71 - 3.12) | 0.24 | 1.27 (0.66 - 2.44) | 0.47 |
| 32+0 to 36+6 | 12 (19.0) | 105 (21.2) | 0.88 (0.41 - 1.74) | 0.87 | 0.71 (0.36 - 1.36) | 0.30 |
| 37+0 to 40+0 | 8 (12.7) | 70 (14.1) | 0.73 (0.33 - 1.47) | 0.42 | 0.89 (0.42 - 1.92) | 0.78 |
| > 40+0 | 4 (6.3) | 50 (10.1) | 0.61 (0.15 - 1.74) | 0.50 | 0.92 (0.32 - 2.65) | 0.87 |
| **Timing of stillbirth** | | | | | | |
| Antepartum | 39 (61.9) | 302 (60.9) | 1.04 (0.59 - 1.87) | 1.00 | 0.88 (0.51 - 1.55) | 0.67 |
| Intrapartum | 6 (9.5) | 30 (6.0) | 1.63 (0.53 - 4.2) | 0.28 | 1.45 (0.59 - 3.53) | 0.42 |
| Not specified | 18 (28.6) | 164 (33.1) | 0.81 (0.43 - 1.48) | 0.57 | 0.90 (0.51 - 1.60) | 0.73 |
| **Fetal sex** | | | | | | |
| Female | 36 (57.1) | 294 (59.3) | 0.92 (0.52 - 1.63) | 0.79 | 1.26 (0.74 - 2.15) | 0.39 |
| Male | 26 (41.0) | 202 (40.7) | 1.02 (0.58 - 1.80) | 1.00 | 0.75 (0.44 - 1.29) | 0.30 |

Table 2S Details on stillbirths in Bavaria during the second lockdown compared to the corresponding period from 2010-2019.
 OR Odds ratio; CI Confidence interval
