# Supplementary material for: Lack of evidence for effects of lockdowns on stillbirth rates during the SARS-CoV-2 pandemic in Bavaria: analysis of the Bavarian perinatal survey from 2010 to 2020
Source: Arch Gynecol Obstet. 2022 Nov 9;308(5):1457–62. doi: 10.1007/s00404-022-06838-0 (PMC9643984; doi:10.1007/s00404-022-06838-0)
Supplement: Supplementary file 3 — Supplementary file3 (DOCX 17 KB) [file 404_2022_6838_MOESM3_ESM.docx]

| **Year** | **Total number of live births** | **Total number of miscarriages** | **Miscarriage < 7+0** | **Miscarriage 7+0– 8+6** | **Miscarriage 9+0 – 10+6** | **Miscarriage 11+0 – 13+6** | **Miscarriage 14+0 – 17+6** | **Miscarriage 18+0 – 20+6** | **Miscarriage 21+0 – 23+6** |
| --- | --- | --- | --- | --- | --- | --- | --- | --- | --- |
| 2020 | 128764 | 12 487 | 1115 (8,9) | 4849 (38,8) | 3899 (31,2) | 2069 (16,6) | 252 (2,0) | 119 (1,0) | 89 (0,7) |
| 2019 | 128227 | 11 959 | 1171 (9,8) | 4428 (37,0) | 3733 (31,2) | 2091 (17,5) | 279 (2,3) | 103 (0,9) | 97 (0,8) |
| 2018 | 127616 | 11600 | 1115 (9,6) | 4145 (35,7) | 3737 (32,2) | 2065 (17,8) | 226 (1,9) | 123 (1,1) | 100 (0,9) |
| 2017 | 126187 | 11893 | 855 (7,2) | 4294 (36,1) | 3949 (33,2) | 2277 (19,1) | 236 (2,0) | 112 (0,9) | 82 (0,7) |
| 2016 | 125686 | 11291 | 643 (5,7) | 4030 (35,7) | 3777 (33,5) | 2267 (20,1) | 237 (2,1) | 135 (1,2) | 104 (0,9) |
| 2015 | 118228 | 11660 | 655 (5,6) | 4260 (36,5) | 3955 (33,9) | 2260 (19,4) | 232 (2,0) | 127 (1,1) | 83 (0,7) |
| 2014 | 113935 | 11906 | 683 (5,7) | 4535 (38,1) | 3989 (33,5) | 2186 (18,4) | 228 (1,9) | 127 (1,1) | 77 (0,6) |
| 2013 | 109562 | 11823 | 685 (5,8) | 4407 (37,3) | 4004 (33,9) | 2241 (19,0) | 236 (2,0) | 116 (1,0) | 67 (0,6) |
| 2012 | 107039 | 11987 | 720 (6,0) | 4542 (37,9) | 3991 (33,3) | 2241 (18,7) | 208 (1,7) | 129 (1,1) | 80 (0,7) |
| 2011 | 103668 | 12163 | 1004 (8,3) | 4370 (35,9) | 3792 (31,2) | 2446 (20,1) | 228 (1,9) | 147 (1,2) | 104 (0,9) |
| 2010 | 105251 | 11542 | 1188 (10,3) | 3984 (34,5) | 3600 (31,2) | 2299 (19,9) | 213 (1,8) | 112 (1,0) | 81 (0,7) |

*Table 3S Miscarriages by gestational age (weeks + days) (ifb - Staatsinstitut für Familienforschung an der Universität in Bamberg 2021, Statistisches Bundesamt (Destatis) 2022)*
